# Outcomes of whole-body photobiomodulation on pain, quality of life, leisure physical activity, pain catastrophizing, kinesiophobia, and self-efficacy: a prospective randomized triple-blinded clinical trial with 6 months of follow-up

**DOI:** 10.3389/fnins.2024.1264821

**Published:** 2024-01-31

**Authors:** Santiago Navarro-Ledesma, James D. Carroll, Ana González-Muñoz, Patricia Burton

**Affiliations:** ^1^Department of Physiotherapy, Faculty of Health Sciences, University of Granada, Melilla, Spain; ^2^THOR Photomedicine Ltd., London, United Kingdom; ^3^Clinical Medicine and Public Health PhD Program, Faculty of Health Sciences, University of Granada, Av. de la Ilustración, Granada, Spain; ^4^Clínica Ana González, Malaga, Spain

**Keywords:** fibromyalgia, chronic pain, quality of life, psychological factors, kinesiophobia, pain catastrophizing, self-efficacy, photobiomodulation

## Abstract

**Background:**

The management of fibromyalgia (FM) symptoms on a global scale remains a complex endeavor. This study endeavors to assess the impact of whole-body photobiomodulation (PBM) compared to placebo PBM on pain, functionality, and psychological symptoms in individuals afflicted with fibromyalgia.

**Objectives:**

The primary objectives of this research were to conduct a comparative analysis of the effects of whole-body photobiomodulation (PBM) and placebo PBM on pain, functionality, and psychological symptoms in patients suffering from fibromyalgia (FM).

**Methods:**

A total of 42 subjects were recruited from a private care practice for participation in this triple-blinded, placebo-controlled, randomized clinical trial. Participants underwent 12 treatment sessions, and assessments were conducted at various intervals, including baseline (T0), midway through the 12-session treatment (T1), at the completion of the 12 sessions (T2), and follow-ups at 2 weeks (T3), 3 months (T4), and 6 months (T5).

**Results:**

Statistical analysis revealed significant reductions in pain at T2, T3, and T5. Additionally, quality of life exhibited marked improvements after sessions at T1, T2, T3, T4, and T5. Leisure activity also demonstrated statistically significant improvements at T2, T3, T4, and T5. Furthermore, kinesiophobia showed significant differences between groups immediately after treatment at T2, T3, T4, and T5. Self-efficacy, when compared between groups, demonstrated significant differences at T3, T4, and T5 (two weeks after treatment). Lastly, pain catastrophizing exhibited significant differences only at T5.

**Conclusion:**

The findings of this study indicate that whole-body PBM treatment for 4 weeks resulted in significant pain reduction and improved quality of life in individuals suffering from FM. Furthermore, kinesiophobia and self-efficacy demonstrated improvements in both short-term and long-term assessments, while pain catastrophizing showed improvement at the 6-month follow-up. Consequently, whole-body PBM emerges as a promising multifactorial treatment option for FM patients, though further studies are required to validate and strengthen these results.

**Clinical Trial Registration:**Clinicaltrials.gov, NCT0424897.

## 1 Introduction

Fibromyalgia (FM) is a prevailing and complex medical condition, characterized by widespread, persistent pain, heightened sensitivity to stimuli, fatigue, sleep disturbances, cognitive impairments, anxiety, and behavioral disruptions, which are among its most prevalent clinical manifestations (Wolfe et al., 2016). The diagnostic criteria set forth by the American College of Rheumatology (ACR) encompass a variety of parameters, including a minimum pain pressure sensitivity of 4 kg, widespread pain affecting at least 4 out of 5 anatomical regions, consistent pain intensity for a duration of at least 3 months, and specific scores on the Widespread Pain Index (WPI) and Symptom Severity Scale (SSS). The diagnosis of FM remains valid and independent of any coexisting medical conditions in the patient (Wolfe et al., 2016). Renowned experts in the field have recently undertaken a thorough review of the aforesaid diagnostic criteria, thereby introducing a comprehensive approach based on five key dimensions. These dimensions entail: 1. Core diagnostic criterio; 2. Common features typically observed in FM; 3. Frequently occurring medical comorbidities associated with FM; 4. The neurobiological, psychosocial, and functional implications of FM; 5. The conjectured neurobiological and psychosocial mechanisms, risk factors, and protective elements relevant to FM’s etiology and progression (Arnold et al., 2019). By embracing this multi-dimensional perspective, healthcare professionals can gain a more insightful understanding of FM, encompassing diverse facets of its presentation, characteristics, and potential underlying mechanisms. Such a comprehensive evaluation aids in formulating well-informed and tailored approaches to the diagnosis, management, and treatment of FM patients (Fillingim et al., 2014).

Fibromyalgia has a significant impact on global epidemiology, affecting approximately 0.2 to 6.6% of the world’s population. As the foremost cause of chronic and diffuse musculoskeletal pain, it poses a considerable challenge to the general population (Wolfe et al., 2016). This condition predominantly afflicts middle-aged adults, with prevalence ranging from 0.5 to 5% in the general population and up to 17.5% in clinical settings (Mas et al., 2008; Queiroz, 2013). Due to the intricate nature of FM and the complexities involved in its diagnosis, achieving a comprehensive treatment approach that addresses all dimensions of the condition remains a considerable challenge. To date, behavioral cognitive therapy and physical exercise have been the most widely accepted interventions for managing FM. However, there persists a dearth of multifactorial treatments that definitively improve all aspects of FM. In this context, novel therapies like whole-body photobiomodulation (PBM) show promise as a potential intervention for individuals suffering from FM (Yeh et al., 2019; Navarro-Ledesma et al., 2022b).

PBM is a non-invasive light therapy employing effective wavelengths of light ranging from 600 to 1,070 nm, with a fluence (energy density) varying between 1 and 20 J/cm^2^. This range optimizes tissue penetration by capitalizing on the high absorption bands of main tissue chromophores, such as hemoglobin and melanin, at wavelengths below 600 nm. For treating superficial tissue, wavelengths in the range of 600–700 nm are utilized, while long wavelengths in the range of 780–950 nm are employed to address deeper-seated tissues (Karu et al., 2005, 2008; Salehpour et al., 2019).

Scientific studies have demonstrated that PBM triggers stimulation of mitochondria, leading to changes in respiratory chain components (e.g., cytochromes, cytochrome oxidase, and flavin dehydrogenase), ultimately cascading into alterations in transcription and translation processes. As a result, there is an increase in ATP production and cellular energy, leading to improved cellular health (Bortoletto et al., 2004; Benedicenti et al., 2008; Moro et al., 2022; González-Muñoz et al., 2023). This mechanism underpins the potential therapeutic benefits of PBM in mitigating the symptoms of FM and enhancing cellular functionality. In this regard, short-term benefits after using whole-body PBM in Spanish women with FM have been explored, showing positive results in pain, quality of life, pain pressure sensitivity, elastic properties of tissue, kinesiophobia and self-efficacy (Yeh et al., 2019; Navarro-Ledesma et al., 2022a, 2023).

This clinical investigation represents a pioneering effort in exploring the enduring impacts of PBM on diverse aspects of FM, including pain, quality of life, leisure physical activity, pain catastrophizing, kinesiophobia, and self-efficacy. By undertaking this study, we aim to enrich our understanding of potential treatment avenues for individuals afflicted with FM.

A meticulously designed triple-blinded randomized clinical trial was implemented to discern the changes occurring in pain, quality of life, leisure physical activity, pain catastrophizing, kinesiophobia, and self-efficacy among FM patients following a whole-body PBM treatment, and we closely monitored their progress over a comprehensive six-month follow-up period. Furthermore, we endeavor to examine the interrelationships between the aforementioned variables, both in the short and the long term.

Through this comprehensive research endeavor, we aspire to provide valuable insights into the sustained effects of PBM as a therapeutic intervention for FM, thereby enhancing the knowledge base of healthcare practitioners and augmenting treatment options for patients grappling with this complex condition. The triple-blinded randomized clinical trial design ensures an objective assessment, while the longitudinal follow-up enables us to unravel potential correlations and associations between various aspects of FM management. By shedding light on these crucial dimensions, we endeavor to contribute significantly to the field of FM research and enhance the care and well-being of affected individuals.

## 2 Methods

### 2.1 Study design

The present research adopts a triple-blinded randomized clinical trial design, as outlined in the previously published study protocol (Navarro-Ledesma et al., 2022b). Ethical clearance was obtained from the local Ethics Committee at the University of Granada (Approval No. 1044/CEIH/2020). This study adheres to the principles stated in the Declaration of Helsinki and is registered in ClinicalTrials.gov under the identifier NCT04248972. The reporting of this study conforms to the standard protocol items specified in the CONSORT Statement (Moher et al., 2010; Eldridge et al., 2016).

### 2.2 Participants

Forty-two individuals diagnosed with fibromyalgia (FM) were recruited from a private clinical practice for participation in this study. A research assistant meticulously evaluated the eligibility of potential participants against the predefined inclusion criteria. Those who fulfilled the stipulated criteria were included in the study. The study commenced with a total of 44 participants, out of which 42 successfully completed the entire research protocol.

### 2.3 Inclusion criteria

The eligibility criteria for participant inclusion were as follows: (i) To be older than 18 years old (ii) Diagnosis of FM confirmed by a qualified rheumatologist in accordance with the modified 2010/2011 ACR classification criteria (Han et al., 2011).

### 2.4 Exclusion criteria

Participants were excluded from the study if they presented with any condition (neurological, orthopedic, or inflammatory) that could potentially interfere with their ability to respond to study assessments. Additionally, individuals with myofascial syndrome pain or fascial muscle disorders, such as trigger points, were excluded from the research.

### 2.5 Recruitment procedures

Participants were recruited from a private clinic and rehabilitation service located in Malaga, Spain. To expand the potential pool of participants, advertisements were placed on various social media platforms. The physiotherapist responsible for participant recruitment provided detailed information about the study, including the eligibility criteria. Upon providing written informed consent, participants were randomly assigned to receive either active or placebo whole-body treatment. In order to enhance treatment adherence, the administering physiotherapist maintained regular communication with the participants, offering reminders about their scheduled sessions and conducting follow-ups.

### 2.6 PBM therapy program and placebo (study protocol)

**Figure 1:**
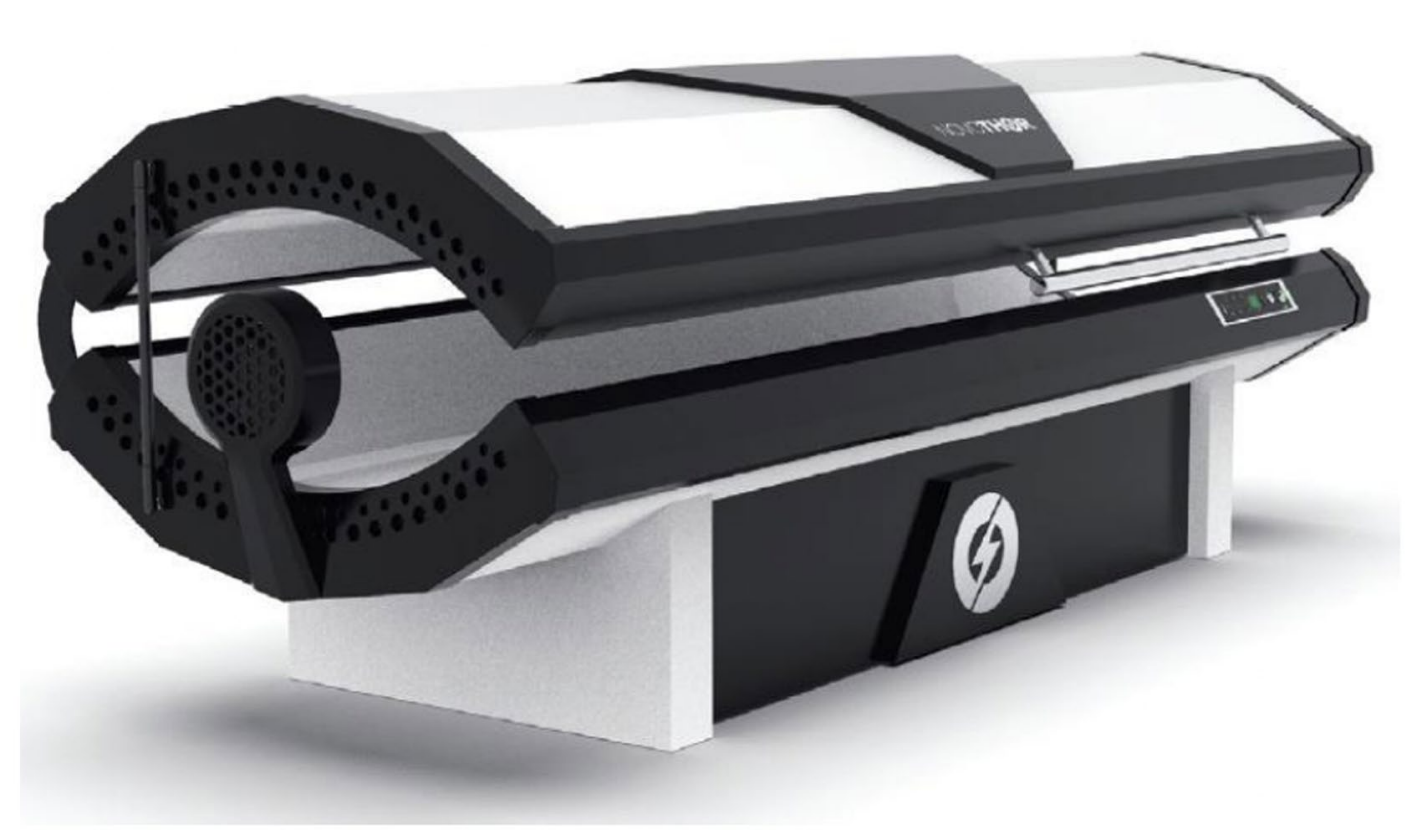
NovoTHOR bed.

Participants received whole-body PBM treatment sessions thrice weekly, spanning a period of 4 weeks, culminating in a total of 12 treatment sessions. During both PBM and placebo interventions, participants assumed a supine position on the treatment bed for 20 min, while adhering to minimal attire requirements. The NovoTHOR® device, a whole-body light bed (see Figure 1 in the previous publication (Navarro-Ledesma et al., 2022b), was employed for administering the treatment. The specific parameters for the PBM intervention are detailed in Table 1.

**Table 1:** NovoTHOR parameters.

NovoTHOR XL parameters		Unit
Wavelengths of red and near-infrared (NIR) LEDs 50:50 ratio	660 850	nm nm
Number of LEDs	2,880	
Power emitted per LED	0.336	W
Beam area per LED (at the lens/skin contact surface)	12.0	cm^2^
Total power emitted	967	W
Total area of NovoTHOR emitting surfaces	34,544	cm^2^
Treatment time	1,200	s
Continuous wave (CW) (not pulsed)	CW	
Irradiance	0.028	W/cm^2^
Fluence	25.2	J/cm^2^

### 2.7 Evaluation of primary and secondary outcome measures

**Figure 2:**
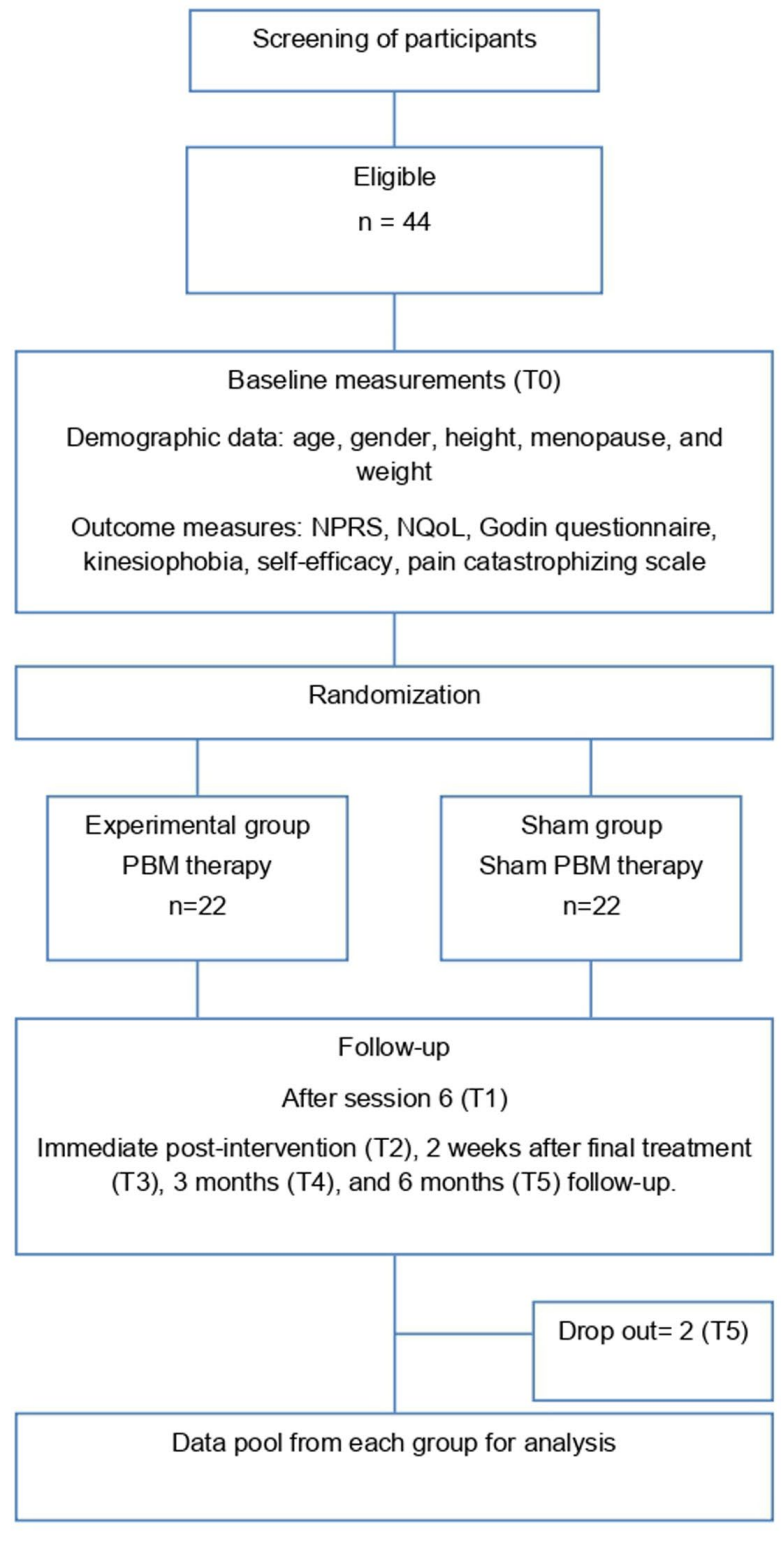
Flow diagram illustrating the assessment times.

The comprehensive assessment of all primary and secondary outcome measures was conducted at various time points throughout the study. Specifically, data collection occurred at baseline (T0), midway through the 12-week treatment protocol after session 6 (T1), immediately after the 12-week full treatment intervention (T2), 2 weeks following the completion of the final treatment session (T3), at the 3-month follow-up (T4), and at the 6-month follow-up (T5), as illustrated in Figure 2.

### 2.8 Outcome measures

#### 2.8.1 Primary outcome measures

1. The Numeric Pain Rating Scale (NPRS) was employed to gauge the intensity of pain experienced by patients. This scale ranges from 0 (indicating “no pain”) to 10 (representing “worst possible pain”). Participants from both groups provided ratings for the average pain intensity they experienced over the preceding 7 days, at each designated measurement point during the study. The NPRS is a well-established tool, demonstrating robust validity and reliability (Jensen et al., 1999).

#### 2.8.2 Secondary outcome measures

1. Health-related quality of life (HRQL) was assessed using the Visual Analog Scale (VAS). The VAS employs a scale ranging from 0 (indicating “no quality of life”) to 10 (signifying “the best possible quality of life”). Participants from both groups rated their average HRQL over the previous 7 days at each assessment point. This scale has shown to be more user-friendly and reliable for patient responses (Shmueli, 2005).
2. The Leisure Time Physical Activity Instrument (LTPAI) was employed to measure the physical activity levels of the patients. This instrument comprises four components, each encompassing three levels of activity: light, medium, and vigorous. Participants reported the number of hours they engaged in each activity level per week over the past 4 weeks, with the total sum indicating their overall physical activity in hours (Jensen et al., 1999). The LTPAI demonstrated satisfactory test–retest reliability for the total score, with an intraclass correlation coefficient (ICC) of 0.86 (95% confidence interval: 0.79–0.93) (Mannerkorpi and Hernelid, 2005).
3. The Pain Catastrophizing Scale was utilized to assess the mechanism through which catastrophizing impacts the pain experience of the patients (Darnall et al., 2017). This validated questionnaire comprises 13 items, divided into three subsections. The scoring scale ranges from 1 to 5, yielding final scores between 0 and 52, where higher scores signify higher levels of catastrophism (Navarro-Ledesma et al., 2022c). The three aspects of the questionnaire are as follows: a. “Helplessness”: Consisting of questions 1 to 5 and 12, this section evaluates the individual’s beliefs regarding their ability to influence their pain experience (Navarro-Ledesma et al., 2022c). b. “Magnification”: Comprising questions 6, 7, and 13, this aspect assesses the extent to which the participant exaggerates the threatening properties of the painful stimulus. c. “Rumination”: Encompassing questions 8 to 11, this subsection evaluates the extent to which the patient is preoccupied with thoughts of pain and unable to escape from such ideation (Navarro-Ledesma et al., 2022c). The Pain Catastrophizing Scale is an extensively validated questionnaire, providing valuable insights into the impact of catastrophism on the pain experience of individuals.
4. The Tampa Scale of Kinesiophobia (TSK) was employed in the study, utilizing the Spanish version. This validated and reliable measure is designed to assess fear of movement (Darnall et al., 2017). The Tampa Scale for Kinesiophobia-11 has demonstrated consistent and reliable performance in evaluating fear of movement among FM patients within clinical settings (Gómez-Pérez et al., 2011; Salvador et al., 2021; Navarro-Ledesma et al., 2022c). It encompasses 11 items, each with four response options ranging from “strongly disagree” (scored as 1 point) to “strongly agree” (scored as 4 points). Consequently, the total score varies from a minimum of 11 to a maximum of 44. Higher scores are indicative of greater fear of movement/injury, signifying elevated levels of kinesiophobia (Larsson et al., 2016; Navarro-Ledesma et al., 2022c).
5. The self-efficacy questionnaire assesses individuals’ personal confidence in effectively accomplishing an activity to achieve the desired outcome (Bandura, 1997). The Self-efficacy scale has been shown to possess adequate psychometric properties, rendering it a valuable tool for health professionals in monitoring patients’ self-efficacy perceptions and guiding the establishment of physical activity and walking exercise intervention goals, as well as their implementation (López-Roig et al., 2021). This questionnaire comprises 10 items, with respondents providing their responses on a four-point scale (MDM Molero Jurado et al., 2019; Navarro-Ledesma et al., 2022c). The final score ranges from 0 to 44, with higher scores indicative of a heightened perception of competence in effectively managing stressful situations.

### 2.9 Statistical analysis

For all data analyses, SPSS® Statistics version 21.0 (IBM, Chicago, IL, United States) was employed. To verify the normality of data distribution, the Shapiro–Wilk test was conducted. Intra-group mean differences across all outcomes at four assessment time points [baseline (T0), after session 6 (T1), immediate post-intervention (T2), 2 weeks after the final treatment (T3), 3 months (T4), and 6 months (T5) follow-up] were examined using repeated-measures analysis of variance (ANOVA). Additionally, to compare the clinical characteristics of the two groups (PBM intervention and placebo groups) at baseline and follow-ups, a multivariate ANOVA (MANOVA) was performed. The MANOVA consisted of six levels, representing each time assessment (T0, T1, T2, T3, T4, and T5), with the two intervention groups considered as independent factors. A value of p less than 0.05 was considered statistically significant.

To assess the magnitude of the effect between and within groups for all quantitative variables, the Cohen d coefficient was calculated. Effect sizes greater than 0.8 were considered large, around 0.5 were considered moderate, and less than 0.2 were considered small (Cohen, 1988).

### 2.10 Sample size calculation

The sample size for this trial was determined based on previous findings from randomized clinical trials and reviews (Farrar et al., 2001; Dworkin et al., 2008; Bourgault et al., 2015). The anticipated mean difference between the intervention and placebo groups was expected to be 2 points on the Numeric Pain Rating Scale (NPRS), which represents the minimum clinically important difference (Farrar et al., 2001). Considering a standard deviation of 2.0 units on the NPRS, a significance level (α) of 0.05, and a statistical power of 90%, a minimum of 22 patients per group was required to detect the difference between the two groups effectively.

## 3 Results

Sociodemographic characteristics of the sample are shown in Table 2.

**Table 2:** Summary of sociodemographic data of the participants.

	Women with FM (*n* = 44)	
Variable	Mean ± SD / frequency (%)	95% CI
Age (years)	52.83 ± 8.04	[50.33, 55.34]
Height (m)	1.63 ± 0.05	[1.62, 1.65]
Weight (kg)	78.19 ± 18.03	[72.57, 83.81]
BMI (kg/m^2^)	29.32 ± 6.21	[27.38, 31.25]
Menopause status		
Premenopausal	29 (69.05)	
Postmenopausal	13 (30.95)	

Quantitative variables are presented as mean ± standard deviation (SD), while qualitative variables are depicted as frequency (%) in the dataset. CI (confidence interval); BMI (body mass index).

Table 3 provides an overview of the between-group differences in variables at various assessment time points, namely, 3 months (T4), and 6 months (T5) follow-up. To see differences at baseline (T0), after session 6 (T1), immediate post-treatment program (T2), and 2 weeks follow-up (T3) see the short-term results study (Navarro-Ledesma et al., 2022c).

**Table 3:** Between group differences variables, at 3 months (T4) and 6 months (T5) follow-up (95%CI).

	T4 3 Month Follow-up	T5 6 Month Follow-up
VAS Pain (mean)	−1.00 *p* = 0.17 (−3.00; 3.60) −0.53^b^ 0.60^c^	2.00 *p* = 0.001 (0.84; 2.78) 1.16^b^ 0.48^c^
HRQL	−3.03 *P* = <0.001 (−4.00;-2.00) −3.2^b^ 0.29^c^	−2.00 *P* = <0.001 (−3.00;-2.00) −2.35^b^ 0.29^c^
LTPAI	−41.23 *P* = <0.001 (−51.28; −31.19) −2.55^b^ 4.97^c^	−43.00 *P* = <0.001 (−53.00;-33.00) −2.86^b^ 4.59^c^
Kinesiophobia	9.52 *P* = <0.001 (4.78; 14.26) 1.24^b^ 2.34^c^	13.00 *P* = <0.001 (10.00; 18.00) 2.16^b^ 1.86^c^
Self-efficacy	−11.19 *P* = <0.001 (−14.00;-8.00) −2.31^b^ 1.50^c^	−12.00 *P* = <0.001 (−18.00;-10.00) −2.04^b^ 2.05
Pain Catastrophizing	7.00 *p* = 0.05 (−5.45; 14.00) 0.64 3.35	10.00 *p* = 0.006 (4.00;18.00) 0.83 3.61

SE, Size effect; The leisure time physical activity instrument (LTPAI).

^a^ 95% CI.

^b^ Cohen’s d.

^c^ SE difference.

### 3.1 Between group differences in pain and quality of life

**Figure 3:**
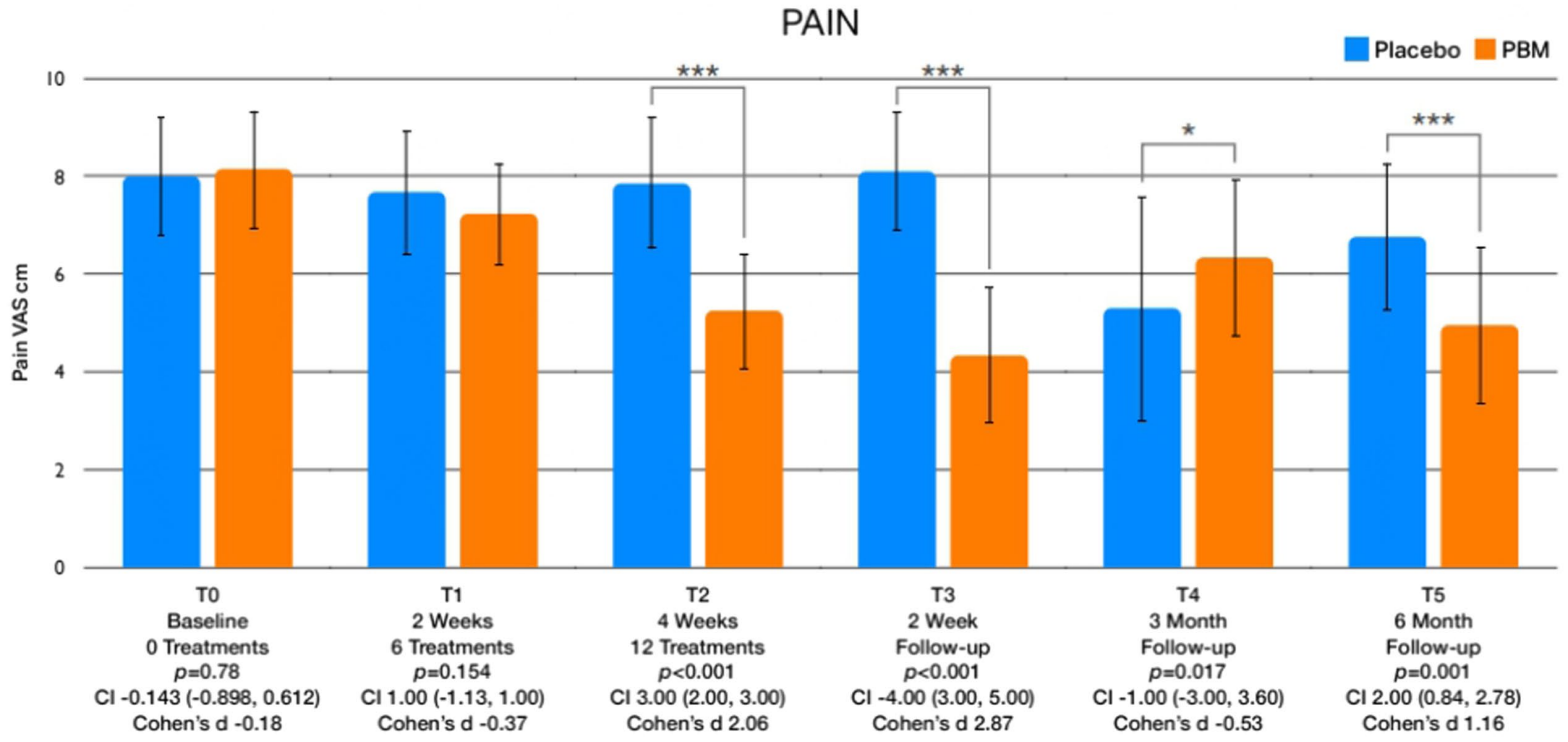
Displays graphical representations depicting the variations in pain levels across different assessment points.

**Figure 4:**
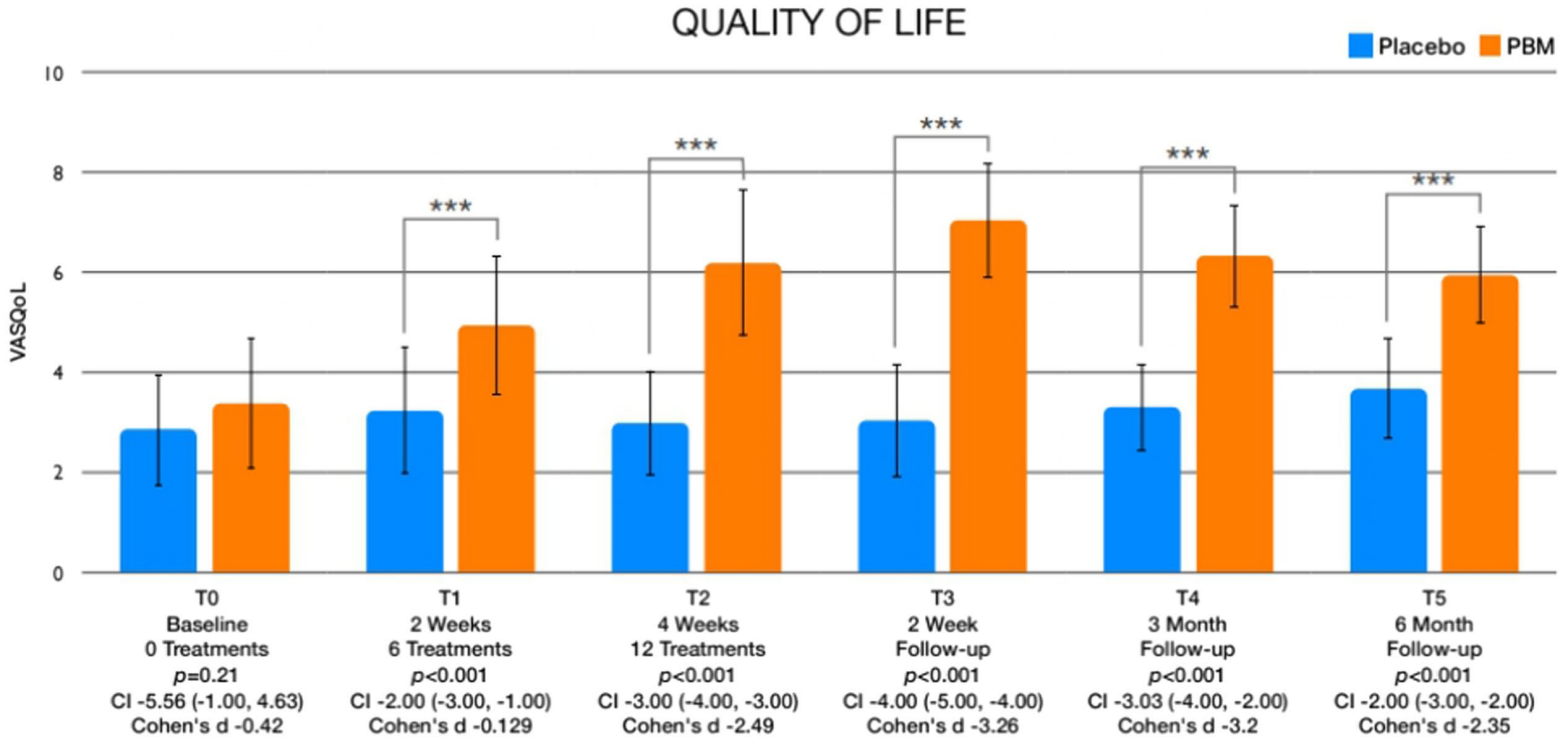
Graphics showing differences in QoL between different assessment times.

The analysis of between-group differences pertaining to pain and quality of life is thoroughly outlined in Table 3, with accompanying visual representations illustrated in Figures 3, 4. Significantly different outcomes were observed in pain levels immediately after treatment (T2), at the 2-week (T3), and 6-month (T5) follow-up periods. In regard to quality of life (Figure 4), statistically significant improvements were evident after session 6 (T1), immediately after treatment (T2), and at the 2-week (T3), 3-month (T4), and 6-month (T5) follow-up assessments.

### 3.2 Between group differences in leisure physical activity and psychological factors

**Figure 5:**
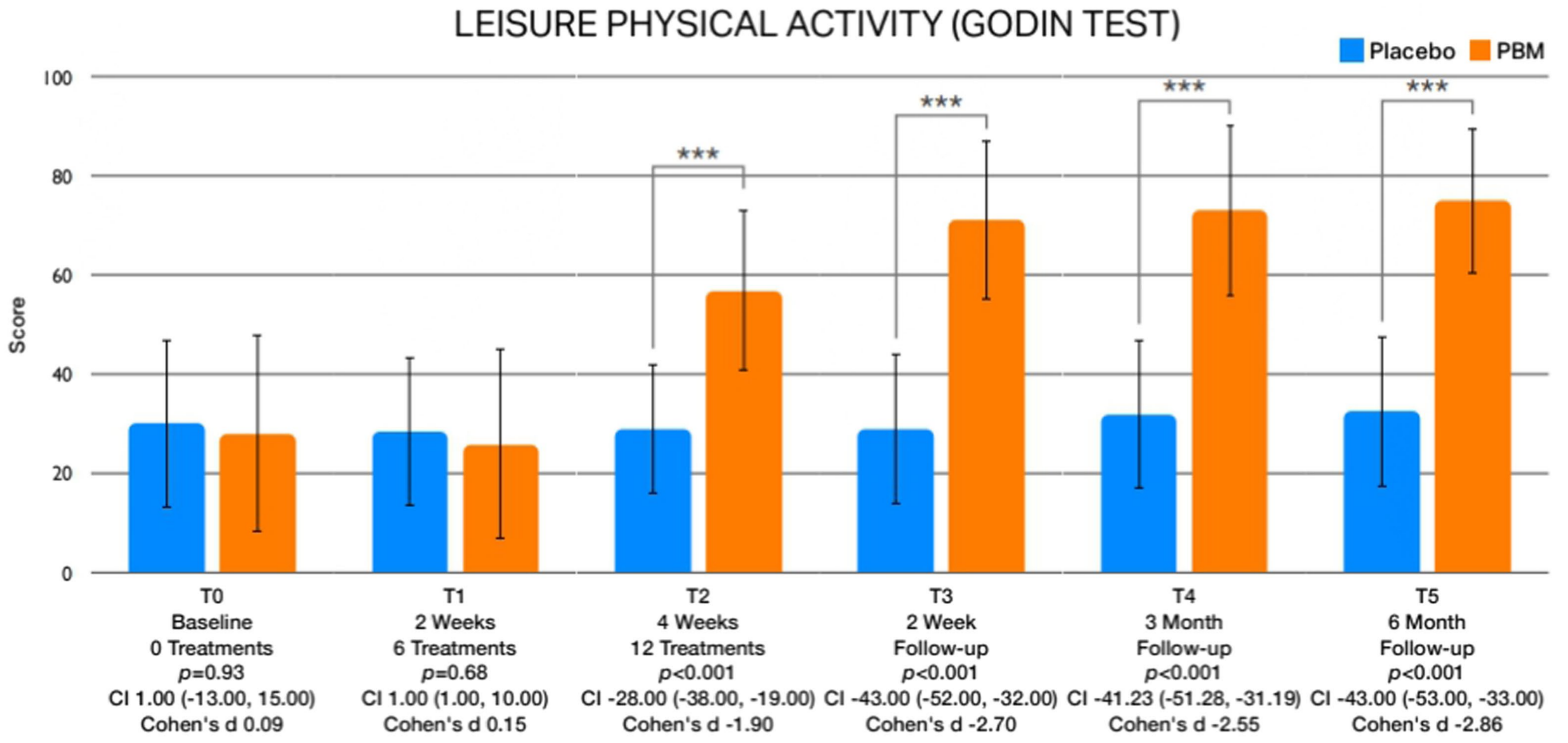
Illustrates the graphical depictions of disparities in leisure physical activity observed at various assessment times.

**Figure 6:**
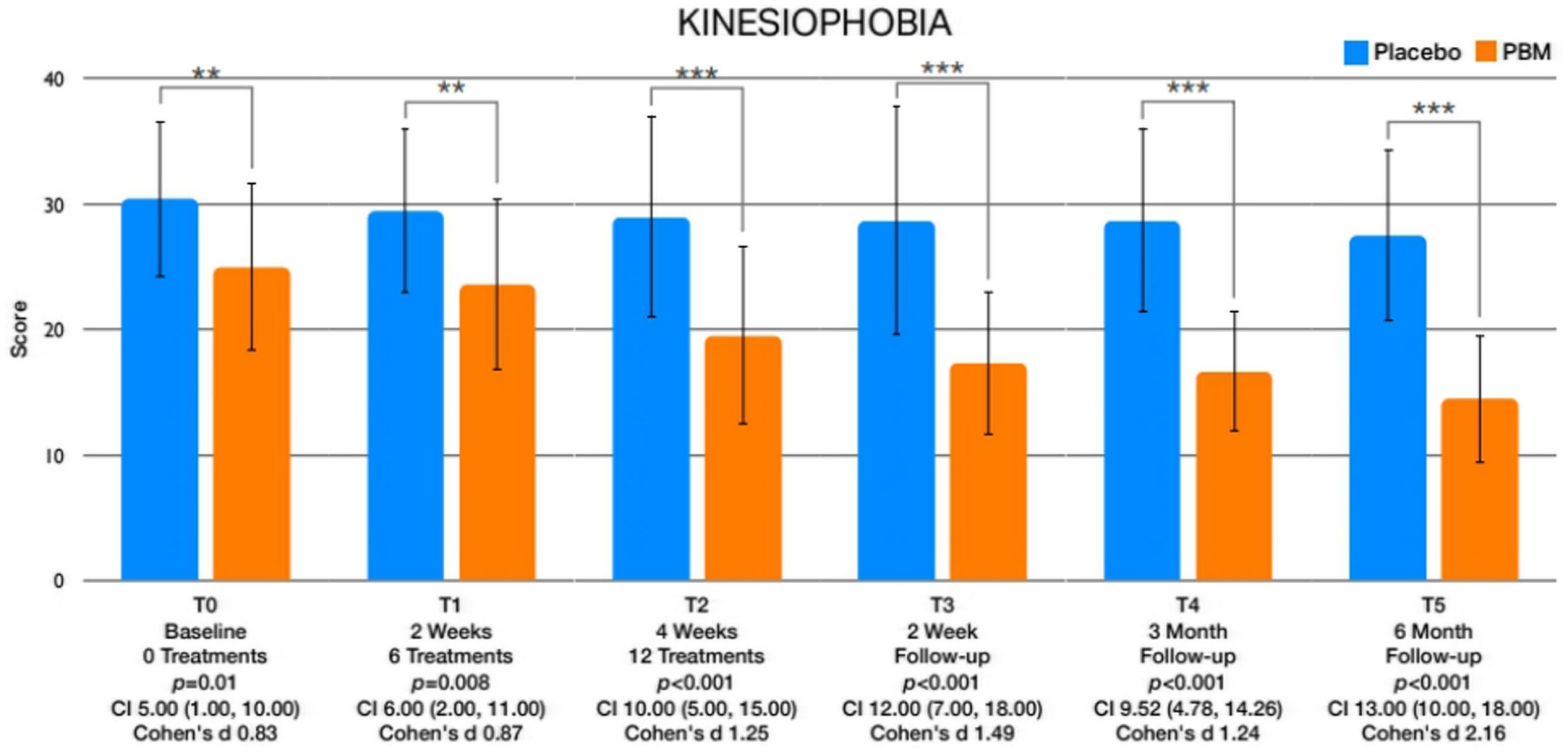
Portrays the graphical representations of the fluctuations in kinesiophobia observed at distinct assessment intervals.

**Figure 7:**
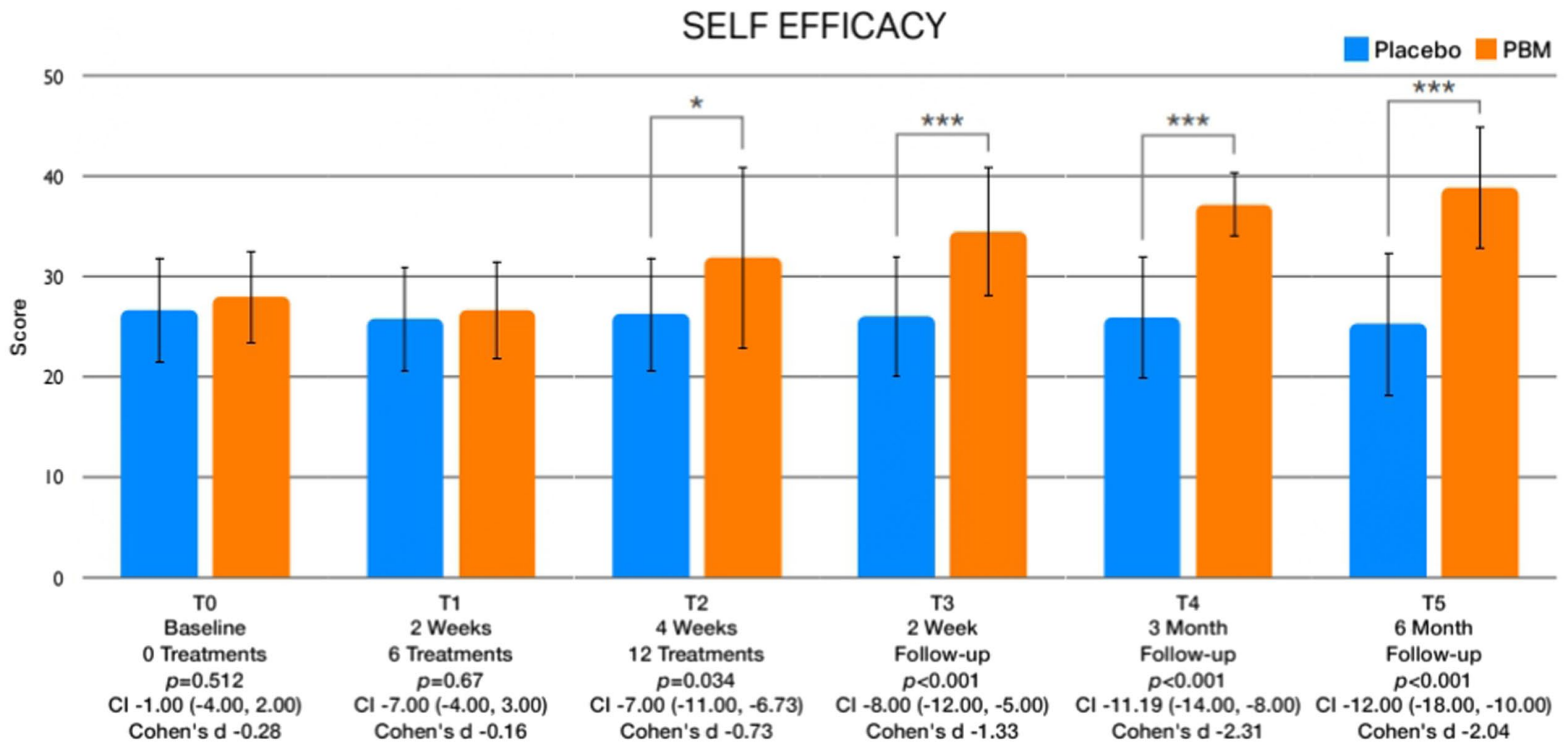
Exhibits the graphical depictions of changes in Self-Efficacy across different assessment times.

**Figure 8:**
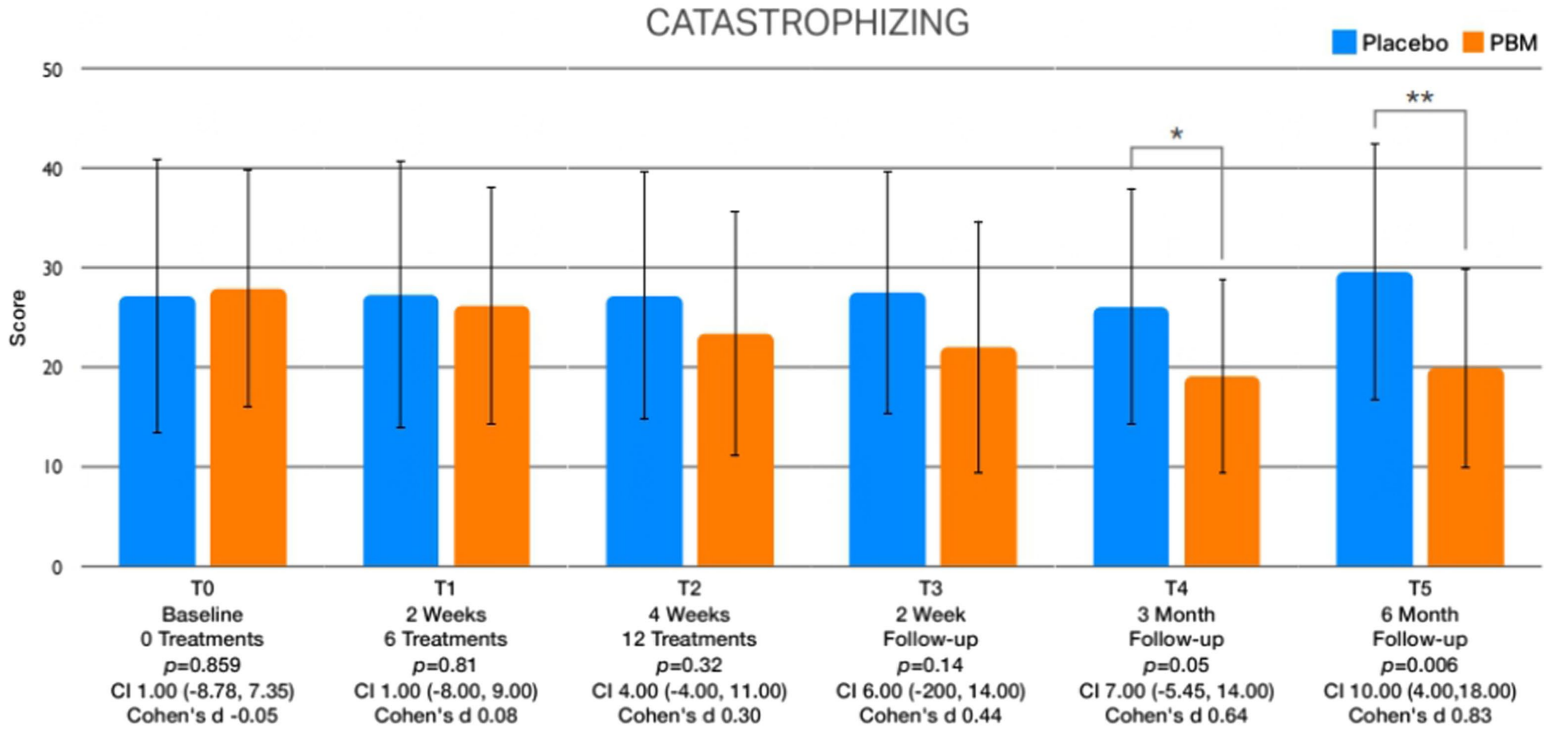
Presents the graphical depictions of variations in pain catastrophizing observed throughout the various assessment points.

The examination of between-group differences in leisure physical activity (Figure 5) and psychological factors is meticulously presented in Table 3. Statistically significant disparities were observed in leisure activity levels immediately after treatment (T2) and at the 2-week (T3), 3-month (T4), and 6-month (T5) follow-up intervals. Regarding psychological factors, noteworthy findings emerged with respect to kinesiophobia, showcasing significant differences between groups immediately after treatment (T2) and at the 2-week (T3), 3-month (T4), and 6-month (T5) follow-up assessments (Figure 6). Similarly, self-efficacy demonstrated significant distinctions between groups at 2 weeks after the treatment (T3), 3 months (T4), and 6 months (T5) follow-up (Figure 7). However, no discernible between-group differences were evident in pain catastrophizing, except at the 6-month follow-up (T5) evaluation (Figure 8).

## 4 Discussion

This placebo-controlled study aimed to explore the effects of a whole-body photobiomodulation (PBM) treatment program on pain, quality of life, leisure physical activity, kinesiophobia, self-efficacy, and pain catastrophizing. The study included a substantial six-month follow-up period to thoroughly assess the long-term impacts of the intervention.

The results of the study revealed noteworthy findings regarding pain reduction, with statistically significant reductions observed after the entire treatment course (T2), as well as at the 2-week (T3) and 6-month (T5) follow-up evaluations. In terms of quality of life, the PBM intervention demonstrated statistically significant improvements after session 6 (T1), immediately after treatment (T2), and at the 2-week (T3), 3-month (T4), and 6-month (T5) follow-up assessments. Moreover, the study documented considerable increases in leisure physical activity levels, with statistically significant improvements observed immediately after treatment (T2), and at the 2-week (T3), 3-month (T4), and 6-month (T5) follow-up points. Regarding kinesiophobia, statistically significant differences were evident between the intervention and control groups, and these differences were observed immediately after treatment (T2), and at the 2-week (T3), 3-month (T4), and 6-month (T5) follow-up assessments. Additionally, self-efficacy exhibited significant variations between the two groups, with notable differences observed 2 weeks after the treatment (T3), and at the 3-month (T4) and 6-month (T5) follow-up evaluations. Lastly, the comparison of pain catastrophizing scores indicated significant differences at the 6-month follow-up assessment (T5).

To our knowledge, this is the first study showing long-term results of whole-body PBM in a population suffering from FM, thus comparison with other studies is difficult. Studies showing the effects of PBM on different conditions have been published, as in oral mucositis (MASCC/ISOO et al., 2020), central nervous diseases (Yang et al., 2021), musculoskeletal conditions such as fractures (Neto et al., 2020), knee osteoarthritis (Vassão et al., 2021), neck disability and chronic neck pain (Gross et al., 2013), pain and function in tendinopathy (Tripodi et al., 2021) and myofascial temporomandibular disorder (Sobral et al., 2021; Yang et al., 2021). Nevertheless, not all studies have found positive effects when using a PBM approach, as in Tomazoni et al. (2020), who found that local PBM did not improve pain and disability in patients suffering from non-specific low back pain, or Ghigiarelli et al. (2020) who did not find significant improvement in a sport population when assessing performance.

Our study demonstrated significant changes in favour of whole body PBM in pain, quality of life, leisure physical activity, kinesiophobia, self-efficacy and pain Interestingly, our results showed a decrease in pain values in both groups at the three-month follow-up. This could be explained by the small, yet existent, increase in the amount of physical activity developed by all participants. On the other hand, it is worth mentioning the significance of the pain reduction from T0 to T5 within the placebo group to fully understand the impact of PBM treatment.

Although differences in pain scores exist between the groups, the placebo group also exhibited decreased values over time. We hypothesize that this may have occurred due to the effect of the whole-body PBM placebo and also because participants had to remain lying, calm, and with closed eyes for 20 min. This might act as a trigger to activate the autonomic nervous system, specifically the vagus nerve, and thereby modulate the central nervous system, ultimately affecting pain. PBM has been shown to stimulate and activate mitochondria metabolism, leading to an increase in ATP production, and a decrease in both oxidative stress and reactive oxygen species. In addition, enhanced nitric oxide synthesis due to the stimulation of cytochrome c oxidase has been shown (Passarella et al., 1984; Poyton and Ball, 2011). Transcranial PBM has been also postulated to stimulate and modulate the central nervous system, and consequently the peripheral nervous system, speculated to be key in the improvement of both central and peripheral sensitization symptoms. In this context, it is well-established that FM is considered a central sensitization syndrome, wherein alterations in neurotransmitters, combined with abnormalities in the ascending and descending pain pathways, including involvement of the hypothalamic–pituitary axis, have been evidenced (Navarro-Ledesma et al., 2022d). The interplay between chronic pain, psychological factors, and the dysregulation of the autonomic nervous system is widely acknowledged and accepted in the scientific community (Holstege, 2016; Navarro-Ledesma et al., 2022e). This includes postural orthostatic tachycardia, greater heart rate increase, and finally an altered blood pressure. In support of this, a recent study showed altered circadian blood pressure variations (Navarro-Ledesma et al., 2022d). In individuals with chronic multifunctional disorders such as FM, there exists an established association between changes in the gut microbiome and elevated glutamate levels, impacting the central nervous system and pain signaling (Wang et al., 2022; Rusch et al., 2023). These alterations in turn can lead to disturbances in gut dysbiosis, heightened gut permeability, and disruptions in mitochondrial function within immune and glial cells (Anderson and Maes, 2020; Wang et al., 2022). The reduction in gut microbiome-derived butyrate plays a role in dysregulating mitochondrial function, both directly and indirectly via increased ceramide levels and decreased melatonin and vagal function (Anderson and Maes, 2020; Casanova et al., 2023). Consequently, dysregulation of circadian rhythms occurs, affecting the resetting of mitochondrial function in immune and glial cells across the circadian rhythm, as previously evidenced in individuals with chronic fatigue syndrome (Anderson and Maes, 2020). PBM has been postulated to modulate the microbiome and this pathway may also explain the changes obtained in this study. However, this is only a hypothesis since metabolomic or microbiome analyses were not performed. Mitochondrial dysfunctions can lead to adverse health effects. In the contemporary context, a variety of illnesses are now associated with issues in mitochondrial function, spanning from disorders in the respiratory, urinary, and metabolic systems to those affecting the nervous and proliferative aspects of health. Comprehending the diverse elements of mitochondrial dynamics and their impact on well-being and illness allows for the implementation of precise and targeted interventions, such as overall-body Photobiomodulation (PBM). Given the pivotal role mitochondria play in health and disease, preserving or restoring mitochondrial function could reasonably be viewed as a fundamental preventive and therapeutic approach (Rusch et al., 2023). Our hypothesis suggests that mitigating risk factors and consequences is feasible, particularly by influencing the transition from oxidative phosphorylation (OXPHOS) to glycolysis guided by mTOR. The persistent Warburg effect contributes to states of proliferation, inflammation, or fibrosis, heightening intracellular biomass. Overall-body PBM, being a low-risk and economically sound strategy that enhances human molecular responses via mitochondrial mechanisms, emerges as a potent tool for promoting mitochondrial health through multiple avenues.

Recognizing the indispensable role of mitochondrial function in understanding health and disease, employing interventions that safeguard or restore normal mitochondrial operation may yield extensive health advantages and prove effective in preventing both primary and secondary chronic diseases, such as fibromyalgia (FM), being these of great importance for society, healthcare professionals and the health system. This approach supports immune system efficiency, an anti-inflammatory state, and adaptability in metabolic and neurological functions. Steering clear of glycolytic states in metabolism and favoring oxidative phosphorylation, as seen in fasting (an anti-Warburg strategy), holds particular significance. Although the mechanisms underlying each overall-body PBM strategy are not fully elucidated, further investigation is warranted, encompassing not only laboratory and animal studies but also clinical trials involving human subjects.

### 4.1 Strengths and limitations of the study

The present study boasts several noteworthy strengths that warrant emphasis. It stands as the inaugural investigation to illuminate the long-term effects of whole-body photobiomodulation in individuals afflicted with FM. Notably, there were no instances of missing data during the treatment phase or the subsequent follow-up assessments. The study design, being a triple-blinded trial, adds to the robustness of the results, effectively reducing potential biases.

Nevertheless, certain limitations warrant acknowledgment. The sample size calculation was based on the primary outcome, thereby not encompassing consideration of other variables. Moreover, age and menopause status may influence the results, as well as differences in kinesiophobia scores at baseline between groups, and this should be taken into account when interpreting the results. Given the novelty of this long-term clinical trial exploring the effects of whole-body PBM in FM subjects, a cautious approach is essential when interpreting the findings, avoiding unwarranted extrapolation to other populations.

### 4.2 Future research

Future research endeavors in the treatment of fibromyalgia (FM) should consider incorporating a combination of pain education, physical activity, and whole-body photobiomodulation (PBM) programs to comprehensively assess changes in pain, quality of life, and psychological factors. Additionally, investigating stress and circadian rhythms through biomarkers like cortisol and melatonin, as well as exploring glucose and insulin metabolism and the composition of the gut microbiota, its metabolites, and relevant nutritional and lifestyle factors in individuals with FM before and after treatment, would be of substantial interest.

Moreover, there is a pressing need for more longitudinal studies to thoroughly analyze the long-term effects of PBM in FM subjects, as well as in other populations experiencing chronic pain.

## 5 Conclusion

The use of NovoTHOR whole-body PBM has demonstrated significant pain reduction and improvements in the quality of life for individuals with FM, both in the short and long term. Additionally, notable enhancements have been observed in psychological factors, such as kinesiophobia and self-efficacy, over the short and long-term, with pain catastrophizing displaying improvement at the 6-month follow-up assessment. However, further research is indispensable to validate our findings and gain deeper insights into the underlying mechanisms driving these effects.

## Data availability statement

The raw data supporting the conclusions of this article will be made available by the authors, without undue reservation.

## Ethics statement

The present study has received ethical approval by the Ethics Committee of Human Research of the University of Granada, Spain (1,044/CEIH/2020). The studies were conducted in accordance with the local legislation and institutional requirements. The participants provided their written informed consent to participate in this study.

## Author contributions

SN-L: Conceptualization, Data curation, Formal analysis, Investigation, Methodology, Project administration, Supervision, Validation, Visualization, Writing – original draft, Writing – review & editing. JC: Conceptualization, Investigation, Resources, Supervision, Validation, Writing – review & editing. AG-M: Conceptualization, Data curation, Investigation, Methodology, Resources, Supervision, Writing – original draft, Writing – review & editing. PB: Conceptualization, Investigation, Methodology, Resources, Supervision, Validation, Writing – original draft, Writing – review & editing.
